# The Effect of Cognitive Function Health Care Using Artificial Intelligence Robots for Older Adults: Systematic Review and Meta-analysis

**DOI:** 10.2196/38896

**Published:** 2022-06-28

**Authors:** Hocheol Lee, Min Ah Chung, Hyeji Kim, Eun Woo Nam

**Affiliations:** 1 Healthy City Research Center Yonsei University Wonju Republic of Korea; 2 Yonsei Global Health Center Yonsei University Wonju Republic of Korea; 3 Center of Evidence Based Medicine Institute of Convergence Science Yonsei University Wonju Republic of Korea; 4 Department of Health Administration College of Software and Digital Healthcare Convergence Yonsei University Wonju Republic of Korea

**Keywords:** older adult population, older adults, cognition, cognitive function, artificial intelligence, socially assistive robots, AI SAR, social prescription, dementia, social support, aging, caregiver, caregiving, meta-analysis, review, Cochrane collaboration, assistive robot, assistive technology

## Abstract

**Background:**

With rapidly aging populations in most parts of the world, it is only natural that the need for caregivers for older adults is going to increase in the near future. Therefore, most technologically proficient countries are in the process of using artificial intelligence (AI) to build socially assistive robots (SAR) to play the role of caregivers in enhancing interaction and social participation among older adults.

**Objective:**

This study aimed to examine the effect of intervention through AI SAR on the cognitive function of older adults through a systematic literature review.

**Methods:**

We conducted a meta-analysis of the various existing studies on the effect of AI SAR on the cognitive function of older adults to standardize the results and clarify the effect of each method and indicator. Cochrane collaboration and the systematic literature review flow of PRISMA (Preferred Reporting Item Systematic Reviews and Meta-Analyses) were used on original, peer-reviewed studies published from January 2010 to March 2022. The search words were derived by combining keywords including Population, Intervention, and Outcome—according to the Population, Intervention, Comparison, Outcome, Time, Setting, and Study Design principle—for the question “What is the effect of AI SAR on the cognitive function of older adults in comparison with a control group?” (Population: adults aged ≥65 years; Intervention: AI SAR; Comparison: comparison group; Outcome: popular function; and Study Design: prospective study). For any study, if one condition among subjects, intervention, comparison, or study design was different from those indicated, the study was excluded from the literature review.

**Results:**

In total, 9 studies were selected (6 randomized controlled trials and 3 quasi-experimental design studies) for the meta-analysis. Publication bias was examined using the contour-enhanced funnel plot method to confirm the reliability and validity of the 9 studies. The meta-analysis revealed that the average effect size of AI SAR was shown to be Hedges *g*=0.43 (95% CI –0.04 to 0.90), indicating that AI SAR are effective in reducing the Mini Mental State Examination scale, which reflects cognitive function.

**Conclusions:**

The 9 studies that were analyzed used SAR in the form of animals, robots, and humans. Among them, AI SAR in anthropomorphic form were able to improve cognitive function more effectively. The development and expansion of AI SAR programs to various functions including health notification, play therapy, counseling service, conversation, and dementia prevention programs are expected to improve the quality of care for older adults and prevent the overload of caregivers. AI SAR can be considered a representative, digital, and social prescription program and a nonpharmacological intervention program that communicates with older adults 24 hours a day. Despite its effectiveness, ethical issues, the digital literacy needs of older adults, social awareness and reliability, and technological advancement pose challenges in implementing AI SAR. Future research should include bigger sample sizes, pre-post studies, as well as studies using an older adult control group.

## Introduction

Population aging is progressing worldwide due to the development of medical technology, and it is predicted that the number of older adults aged ≥65 years will increase from 730 million in 2019 to 1.5 billion in 2050 [1]. The World Health Organization has marked dementia and the mental health of older adults as public health problems due to an increase in the older adult population [2]. Dementia is a disease that occurs mainly in older adults aged ≥65 years and causes cognitive dysfunction, hyperactivity, sleep disturbance, violence, and depression, weakening daily life activities and making social activities difficult [3,4]. Currently, most patients with dementia are receiving treatment through drug therapy, but the medication rate is low since the symptoms of dementia impair the patients’ ability to recognize the need to take medication [5]. To overcome these problems, treatment methods that combine nonpharmacological treatment with drug treatment are increasing. Psychosocial therapy is being used as a representative nonpharmacological treatment for the improvement of cognitive function of older adults around the world. The United Kingdom’s National Health Service is implementing social prescribing, a nonpharmacological intervention program that connects patients with mental health conditions including dementia with nonmedical support sources in the community. Representative social prescribing programs include line dance, gardening, art therapy, music therapy, counseling therapy, and caring therapy [6]. According to previous studies, treatment methods based on interaction and conversation, rather than medication, for older adults with weakening cognitive function provide a sense of relief and stability, which in turn increases emotional support and social communication and thereby helps them recover their cognitive function [7]. As the older adult population increases, so does the population of older adults with cognitive impairment, and as a result, human resources and various nonpharmacological treatment programs are required. However, due to the rapidly aging global population, there is a shortage of caregivers; caregivers are particularly reluctant to take care of older adults with dementia due to mental stress, and the number of caregivers for patients with dementia is decreasing. As an alternative solution to this problem, technologically proficient countries such as the United States, Korea, Japan, and Australia are prioritizing the development of artificial intelligence (AI) socially assistive robots (SAR) as a part of digital health care [8]. According to previous studies, AI SAR have been found to be effective in preventing the overwork of caregivers for older adults, increasing work efficiency, and performing 24-hour monitoring [9,10].

AI SAR are robots designed to interact with humans (eg, older adults) using AI. As a method of promoting interaction and social participation among older adults, the development and research of AI SAR are actively being conducted [11,12]. AI SAR started in the form of an animal-type pet robot in early development and have been developed into various forms such as human- and doll-like robots. Regardless of the form, AI SAR were found to effectively increase the frequency of independent communication by making older adults initiate conversations [13]. Due to the development of various technologies, AI SAR have developed to the extent in which they can interpret and express not only verbal expressions, gestures, eye contact, and emotional expressions but also nonverbal communication methods, and their ability to communicate with older adults is also developing at an increasing rate. The role of AI becomes more important particularly when an infectious disease such as COVID-19 becomes prevalent, which limits the visiting service of nursing personnel.

AI SAR have been proven to be effective in enhancing interaction [14,15], improving the quality of life [16], improving depression and anxiety [17], and improving the quality of life of patients with dementia [18] for older adults aged ≥65 years. In addition, there has been a meta-analysis study published on the effect of the use of robots on older adults aged ≥65 years [19]. However, in an effectiveness study through a meta-analysis of AI SAR, it was confirmed that the study results including agitation, depression, and quality of life [19-21], etc, were inconsistent depending on the intervention method, SAR method, and characteristics of the older adults. A meta-analysis is necessary to standardize these various results, methods, and indicators. In other words, although the intervention using AI SAR has various effects on older adults, which has been proven through various studies, a meta-analysis based on the results of existing studies is necessary to clarify what kind of effect each indicator has. Currently, there is a lack of meta-analysis studies that analyze the effect of robots on cognitive function by setting a control group.

Therefore, the purpose of this study was to understand the effect of intervention using AI SAR on the cognitive function of older adults through a systematic literature review. To this end, the detailed goals were as follows: (1) to search and review the existing literature on the effect of AI personal care on cognitive function; (2) to objectively identify the feasibility of the effect of nursing care service through AI SAR and the effect of AI SAR on cognitive function based on the results of the collected theses; and (3) to provide the basis for supporting policies and research on providing AI SAR to older adults aged ≥65 years.

## Methods

### Study Design

This systematic literature review and meta-analysis study identified the intervention effect of AI SAR to understand its effect on the cognitive function of older adults aged ≥65 years.

### Search Strategy

This study was conducted according to the systematic literature review method by the Cochrane collaboration and the systematic literature review flow of PRISMA (Preferred Reporting Items for Systematic Reviews and Meta-Analyses) [22,23]. The target data included original, peer-reviewed studies published from January 2010 to March 2022. The databases used for the search included PubMed and Google Scholar.

The search words were derived by combining keywords including Population, Intervention, and Outcome according to the Population, Intervention, Comparison, Outcome, Time, Setting, and Study Design (PICOTS-SD) principle (Multimedia Appendix 1).

- Population: “Elderly” OR “Elderly People” OR “older adults” OR “older people” OR “senior” OR “Dementia” OR “Alzheimer” OR “Cognitive impairment”
- Intervention: “Robot” OR “AI robot” OR “social assistive robot” OR “social interactive robot” OR “assistive robot” OR “companion robot” OR “robot interaction” OR “health care robot”
- Outcome: “MMSE” OR “Mini-Mental State Examination” OR “cognitive function” OR “cognitive” OR “cognitive impairment” OR “cognitive disorder” OR “mental health”

### Eligibility

This study used the PICOTS-SD selection and constituted the question “If older adults aged ≥65 years are provided with AI SAR, what would be the effect on cognitive function in comparison with a control group?” The PICOTS-SD criteria for this question includes older adults aged ≥65 years (Population), AI SAR (Intervention), comparison group (Comparison), popular function (Outcome), and prospective study (Study Design). Subsequently, a systematic literature review was conducted, focusing on the core research.

From the above PICOTS-SD criteria, studies in which even one condition among subjects, intervention, comparison, and study design was different than those indicated were excluded from the literature review.

### Quality Assessment

To minimize the deviation that occurs in literature search, 2 researchers searched and collected the data and then confirmed whether the same results were obtained. In addition, only peer-reviewed studies were included to increase the validity of the literature selection.

A risk of bias (ROB) assessment was performed to evaluate the quality of the literature selected in this study. Both subjective and objective evaluations were performed in the ROB assessment. For subjective evaluation, Cochrane ROB assessment was used [22]. Cochrane ROB assessment consisted of (1) Random Sequence Generation, (2) Allocation Concealment, (3) Blinding of Outcome Assessment, (4) Incomplete Outcome Data, (5) Selective Reporting, and (6) Other Bias, and the researchers confirmed that the studies were selected according to the guidelines. Subjective evaluation was conducted using a funnel plot.

All studies were reviewed by 3 researchers and selected based on a consensus of opinions to confirm the validity and consistency of the study.

### Data Extraction and Data Synthesis

In this study, data were extracted and processed for the analysis of the selected studies. Data were synthesized by entering into Excel the (1) characteristics of the literature (year, journal, author, country, and study design), (2) research method (intervention, number of experimental groups, and number of control groups), and (3) research results (mean and SD of the experimental group and control group).

### Data Analysis

This study calculated the effect size from 9 studies to analyze the effect of AI SAR on the cognitive function of older adults. To calculate the effect size, a normal distribution of the mean of each study was applied using a random effects model. For assigning weights in the random effects model, the DerSimonian and Laird method was used, including between-study variance [24]. For the effect size, the Standardized Mean Difference was used as an analysis value, and 95% CI and inverse of variance were used for weights [25].

To analyze the heterogeneity of the 9 studies investigated in this study, a visual review was conducted using a Forest plot and a Galbraith plot. The effect size, direction, and CI of each study were analyzed using the Forest plot, and they were listed by year, effect size, and sample size. In the Galbraith plot, the effect size divided by the SE was plotted on the y-axis, and the reciprocal of the SE was plotted on the x-axis. If a data point was plotted within 2 SEs on the regression line, then it was interpreted as having no heterogeneity.

To identify the reporting bias of this meta-analysis study, publication bias was classified by analyzing the contour-enhanced funnel plot and determining whether it was symmetrical.

## Results

### Search Result

**Figure 1:**
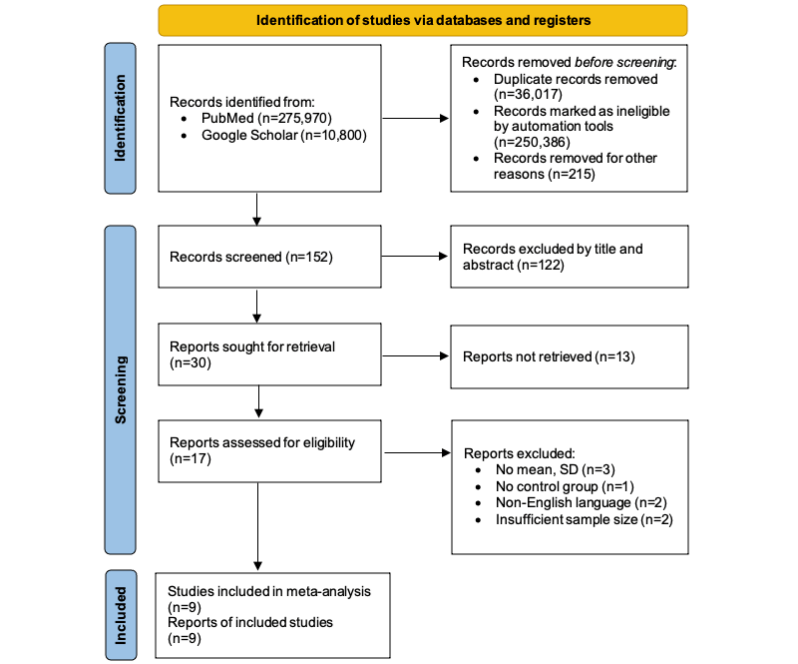
PRISMA (Preferred Reporting Items for Systematic Reviews and Meta-Analyses) flow chart.

In total, 275,970 studies from PubMed and 10,800 studies from Google Scholar were searched using keywords to select the suitable literature for this study. Titles and abstracts were reviewed for 152 studies, excluding duplicate studies (36,017 cases), those marked ineligible by automation tools (250,386 cases), and those removed for other reasons (215 cases). A total of 30 studies were selected as a result, and among them, 9 studies were included in the meta-analysis, excluding those that were not retrieved (13 cases), lacked statistics (3 cases), lacked a control group (1 case), were in a non-English language (2 cases), and had an insufficient sample size (2 cases; Figure 1).

### Characteristics of Studies Included in the Meta-analysis

The characteristics of the 9 studies selected through the PRISMA procedure are shown in Table 1. The selected studies were conducted between January 2010 and March 2022, and they evaluated the effectiveness of AI SAR on cognitive function improvement in older adults aged ≥65 years, using Mini Mental State Examination (MMSE) evaluation and comparison with a control group. A total of 575 individuals aged ≥65 years participated in the selected studies, including 273 in the experimental group and 302 in the control group. Among the selected studies, there were 6 randomized controlled trials and 3 quasi-experimental design studies.

All selected studies used MMSE to measure cognitive function, and other indices were used, including the Global Deterioration Scale (GDS), Neuropsychiatric Inventory (NPI), Apathy Scale for Institutionalized Patients with Dementia Nursing Home version (APADEM-NH), Quality of Life in Late-stage Dementia (QUALID) scale, Apparent Emotion Rating (AER) Instrument, Korean version of the Cohen-Mansfield Agitation Inventory (K-CMAI), Subjective Memory Complaint Questionnaire (SMCQ), Korean version of the Consortium to Establish a Registry for Alzheimer’s Disease (CERAD-K), Geriatric Depression Scale Short Form: Korean Version (GDSSF-K), Japanese version of the Montreal Cognitive Assessment (MOCA-J), Tokyo Metropolitan Institute of Gerontology-Index of Competence (TMIG-IC), Functional Independence Measure (FIM), Duke Older Americans Resources and Services (OARS) Procedures, Mobility subsection of Dysfunction section of Sickness Impact Profile (SIP), and Craig Handicap Assessment and Reporting Technique (CHART).

**Table 1:** Characteristics of the studies included in the meta-analysis.

Author, year	Study design	Sample size (intervention group; control group)	Intervention	Outcome indicator
Tanaka et al, 2012 [26]	Randomized controlled trial	18; 16	Community robot resembling a 3-year-old boy	MMSE^a^ and BMI
Yoshii et al, 2021 [27]	Quasi-experimental design	47; 47	Humanoid robot	MMSE
Valentí Soler et al, 2015 [28]	Randomized controlled trial	33; 38	PARO robot	MMSE, GDS^b^, NPI^c^, APADEM-NH^d^, and QUALID^e^
Valentí Soler et al, 2015 [28]	Randomized controlled trial	30; 38	NAO robot	MMSE, GDS, NPI, APADEM-NH, and QUALID
Koh and Kang, 2018 [29]	Quasi-experimental design	17; 16	PARO robot	MMSE, AER^f^, and K-CMAI^g^
Park et al, 2021 [30]	Randomized controlled trial	45; 45	Humanoid robot (Sil-bot)	MMSE, SMCQ^h^, CERAD-K^i^, and GDSSF-K^j^
Otake-Matsuura et al, 2021 [31]	Randomized controlled trial	32; 33	Photo-integrated conversation moderated by robots	MMSE-J^k^, MOCA-J^l^, GDS-15-J^m^, and TMIG-IC^n^
Oh et al, 2015 [32]	Quasi-experimental design	17; 25	Silver-care robot	MMSE and GDS
Tomita et al, 2007 [33]	Randomized controlled trial	34; 44	X10 ActiveHome kit	MMSE, FIM^o^, OARS^p^, SIP^q^, and CHART^r^

^a^MMSE: Mini Mental State Examination.

^b^GDS: Global Deterioration Scale.

^c^NPI: Neuropsychiatric Inventory.

^d^APADEM-NH: Apathy Scale for Institutionalized Patients with Dementia Nursing Home version.

^e^QUALID: Quality of Life in Late-stage Dementia.

^f^AER: Apparent Emotion Rating.

^g^K-CMAI: Korean version of the Cohen-Mansfield Agitation Inventory.

^h^SMCQ: Subjective Memory Complaint Questionnaire.

^i^CERAD-K: Korean version of the Consortium to Establish a Registry for Alzheimer’s Disease.

^j^GDSSF-K: Geriatric Depression Scale Short Form: Korean Version.

^k^MMSE-J: Japanese version of the Mini Mental State Examination.

^l^MOCA-J: Japanese version of the Montreal Cognitive Assessment.

^m^GDS-15-J: Japanese version of the 15-item Geriatric Depression Scale.

^n^TMIG-IC: Tokyo Metropolitan Institute of Gerontology-Index of Competence.

^o^FIM: Functional Independence Measure.

^p^OARS: Duke Older Americans Resources and Services Procedures.

^q^SIP: Mobility subsection of Dysfunction section of Sickness Impact Profile.

^r^CHART: Craig Handicap Assessment and Reporting Technique.

### Assessment of Publication Bias

**Figure 2:**
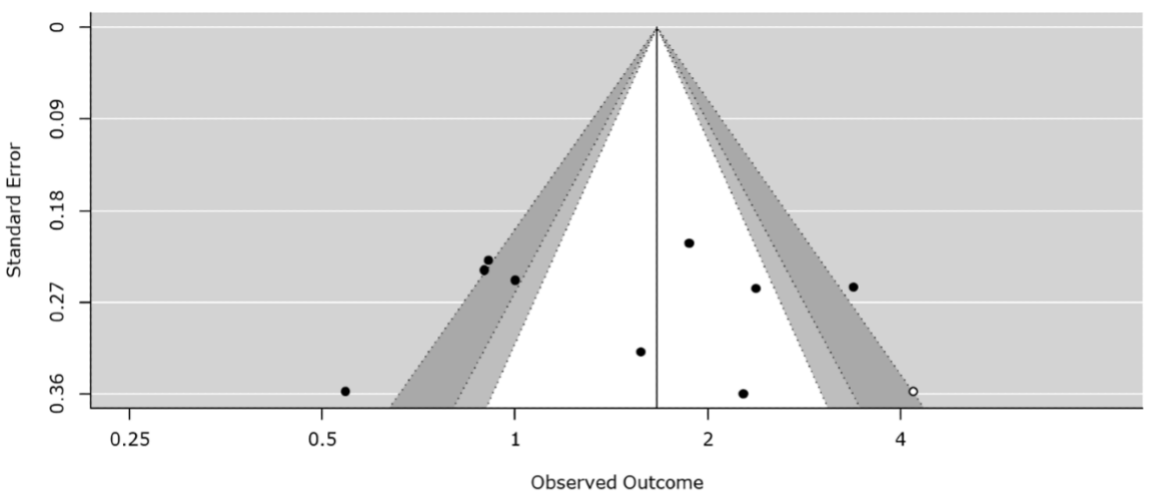
Adjusted funnel plot to examine publication bias.

To secure the reliability and validity of the 9 studies that were selected, publication bias was examined using the contour-enhanced funnel plot method. As a result, it was confirmed that the selected literature in this study represents a well-behaved data set, showing general symmetry (Figure 2).

### Effect Size of AI SAR

**Figure 3:**
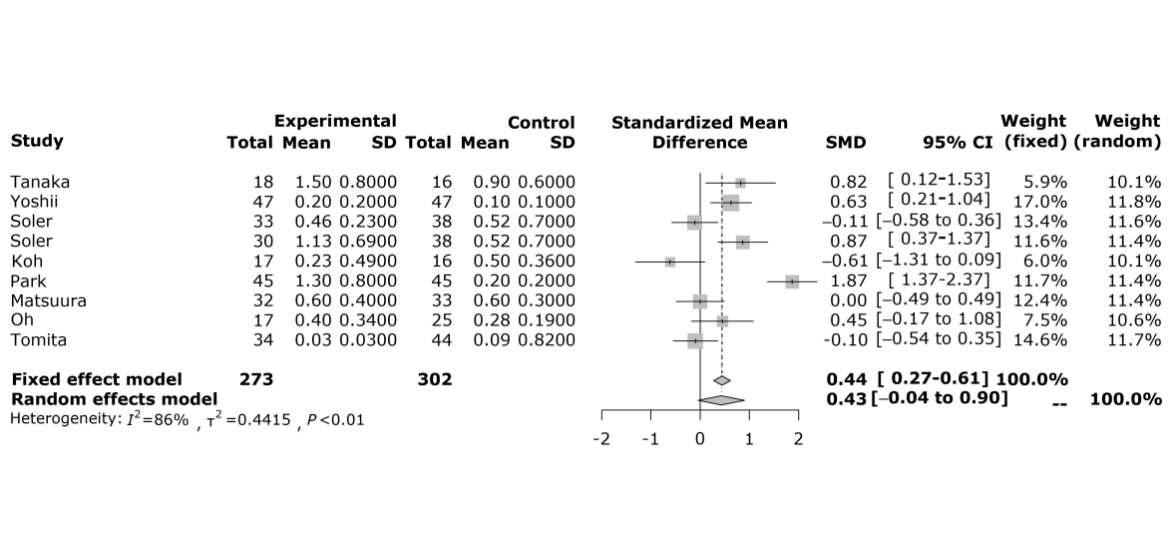
Forest plot results. SMD: standardized mean difference.

**Figure 4:**
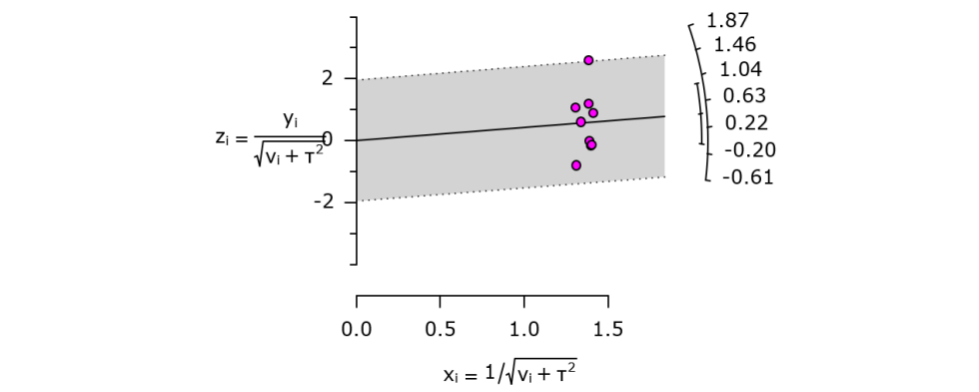
Galbraith plot to identify heterogeneity.

For the 9 studies included in the systemic literature review, the standardized mean differences were calculated by the Hedges *g* formula using the mean, SD, and sample size of the pre-post change of the MMSE indices of the experimental and control groups. This was visualized as a Forest plot (Figure 3). As a result of the meta-analysis, the average effect size of AI SAR was shown to be Hedges *g*=0.43 (95% CI –0.04 to 0.90), indicating that AI SAR are effective in reducing the MMSE scale, which reflects cognitive function. The overall size heterogeneity was confirmed according to the ratio of the interstudy variance to the total variance (*I*^2^=86%; *P*<.001). Furthermore, as a result of confirming the heterogeneity between studies using the Galbraith plot, it was confirmed that all studies had no heterogeneity within the 95% CI as the SEs were within 2 (Figure 4).

## Discussion

### Principal Findings

Due to the aging global population and technological developments, AI SAR for the care of older adults are continuously being developed. The purpose of this study, therefore, was to objectively identify the effect of AI SAR on the cognitive function of older adults through a systematic literature review and prepare and provide recommendations regarding AI SAR policy. The effectiveness of care services using robots in aging populations is socially recognized through continuous research and publications, but many experts agree that more objective evidence is needed. To this end, this study conducted a systematic review and meta-analysis on 9 studies that analyzed the effect of AI SAR on cognitive function improvement in older adults aged ≥65 years since 2010. As a result, it was found that AI SAR showed a significant effect in improving the cognitive function of older adults aged ≥65 years (Hedges *g*=0.43, 95% CI: –0.04 to 0.90). This is consistent with the results of a systematic review article, which states that robots are effective in improving cognitive function in older adults [34]. The difference between previous studies and this study is that the effects of various indicators were confirmed (GDS, NPI, APADEM-NH, QUALID, K-CMAI, SMCQ, CERAD-K, GDSSF-K, TMIG-IC, MOCA-J, FIM, OARS, SIP, and CHART).

In all 9 studies, a control group was designed to analyze the effects. With these results, we will mainly discuss (1) interactive robots, (2) the prospect of resolving the shortage of caregivers, (3) the possibility of expanding the digital social prescription program, and (4) what needs to be overcome for the application of AI SAR.

First, it is essential for AI SAR to be an interactive robot. The 9 studies that have been analyzed have in common that AI SAR could interact with older adults through dialogue. According to previous studies, the cognitive function of older adults aged ≥65 years was shown to be more effective in two-way communication than one-way communication [35]. In this case, the form of the robot greatly affects the formation of rapport. The 9 studies that have been analyzed made use of SAR in the form of animals, robots, and humans. Among them, AI SAR in anthropomorphic form were able to improve cognitive function more effectively. According to a literature review on AI SAR marketing, it is necessary to develop a robot that resembles a human being as much as possible, and it emphasizes the need to develop customized robots for customers by customer segmentation [36]. In this study, it was also found that human-shaped dolls and humanoid forms increased cognitive function more effectively than nonhuman, doll-shaped robots.

Second, as AI SAR have recently been developed to the extent that they can communicate with each other, they have been loaded with various functions including health notification, play therapy, counseling service, conversation, and dementia prevention programs. The development and expansion of AI SAR programs are expected to improve the quality of care for older adults and prevent an overload of caregivers. By conducting a meta-analysis of 9 studies, this study was able to objectively confirm that AI SAR are effective in improving cognitive function. This is evidence that AI SAR can relieve some of the work of caregivers looking after older adult patients with cognitive impairment, including patients with dementia. Older adults living alone with cognitive function impairment particularly require continuous monitoring due to the risk of various incidents when they are alone at home, which demands that caregivers be on-call 24 hours a day. However, since technological advancements have allowed AI SAR to continuously monitor older adults for 24 hours a day and contact facilities in the case of an emergency, it is expected to partially replace the work of caregivers in the future.

Third, AI SAR can be expanded to digital social prescription programs as a nonpharmacological intervention that improves the cognitive function of older adults aged ≥65 years. Social prescription began based on an idea conceived in the 1990s, in which patients were encouraged to exercise as part of their treatment. In the United Kingdom, the National Health Service defines social prescribing as a general practitioner prescribing a nonpharmacological intervention community program to a patient using community resources [37]. Recently, due to the shortage of mental health counselors and caregivers, digital social prescriptions, which convert existing social prescription programs to programs using digital technology, are expanding [38]. Social prescription has conflicted with existing prescription methods for the past 10 years, and there has been a lot of controversy. The main argument is that it is difficult to prove the effectiveness of social prescription, which is a nonpharmacological treatment, unlike existing pharmacological treatments. However, AI SAR are a representative digital social prescription program and a nonpharmacological intervention program in the form of a care service that communicates with the older adults aged ≥65 years 24 hours a day. The data collected through this 24-hour monitoring will be an important stepping-stone in proving that AI SAR are effective as a digital social prescription program. However, more objective research and development is necessary to support this.

Fourth, despite the effectiveness of AI SAR, there are currently problems to be overcome, including (1) ethical issues, (2) the digital literacy needs of older adults, (3) social awareness and reliability, and (4) technological advancements, etc.

The ethical and social issues of AI SAR should be addressed first. The development of AI SAR has replaced some of the existing caregivers, and AI SAR have been developed to a level that can provide care for older adults. However, as they enter the daily life of older adults, personal information is highly likely to be exposed. This is because AI SAR generate and transmit various real-time data using a camera, microphone, and voice tool.

AI SAR are a digital device, and basic digital literacy is required, particularly for charging and the user manual of the device. However, older adults have low digital literacy and limited access to devices, especially in low-income countries, rural areas, and in higher age groups [39]. The low digital literacy of older adults will cause problems in the use of AI SAR. In other words, the digital literacy of older adults is a basic requirement for the application of AI SAR. Therefore, to improve the digital literacy of older adults at a social level, it is necessary to provide a pre-education service to expand the AI SAR service.

Socially, there is a negative view on robots managing various tasks in daily life. Robots took on many tasks as they became gradually more developed and interactive. In the case of AI SAR, they live together with older adults and carry out 24-hour monitoring. Due to this, if a systemic defect causes AI SAR to make a mistake when concerning the older adults, who are a vulnerable group, it is possible that a negative view on the introduction of AI SAR in society might spread. To prevent this, systematic and continuous algorithm development and cognitive training of AI SAR is suggested, including the need to develop an internal algorithm that makes AI SAR apologize for their mistakes [40].

AI SAR still require further technological advancement and have challenges that need to be addressed. Currently, they perform limited word selection and dialogue based on algorithms, and functions such as dementia prevention programs are provided with limited technology. It is clear that the role of AI SAR should gradually expand at a time when the global population is aging, the number of caregivers is decreasing, and technological advancement is becoming essential for solving these issues. To improve the cognitive function of older adults, more development is needed to provide physical care, and technological advancement is necessary to indirectly help them engage in social activities through various communications.

This study possesses some limitations. First, the number of sampled studies that investigated the improvement of cognitive function through SAR was insufficient. It is necessary to conduct future research by including single pre-post studies as well as studies conducted by selecting an older adult control group. Second, only studies using MMSE to measure cognitive function improvement were selected, but various indices such as GDS and NPI also exist. A meta-analysis including all the different indices is recommended for obtaining more objective results in the future. Third, the types of AI SAR used in the 9 selected studies were all different. This is a limitation as it is difficult to measure the nonsampling error that occurs due to the different types of AI SAR. Lastly, we searched using the PubMed and Google Scholar databases. Therefore, we may be missing articles from another database such as IEEE, Embase, and Cochrane Library. In future, we will consider searching using the IEEE, Embase, and Cochrane Library databases.

### Conclusion

In this study, a meta-analysis was performed on 9 studies to examine the effect of AI SAR on improving cognitive function in older adults. As a result, AI SAR were found to be effective in improving cognitive function, suggesting that it is possible to (1) socially expand interactive robots, (2) solve the shortage of caregivers, and (3) expand AI SAR use into a digital social prescription program. Furthermore, the challenges of ethical issues, the digital literacy needs of older adults, social cognition and reliability, and technological development must be solved for the commercialization and expansion of AI SAR. Nonetheless, in times of pandemics such as COVID-19, the need for AI-assisted care is likely to further increase due to its safety.

## Appendix

Multimedia Appendix 1 Search keywords.

## Abbreviations

AER: Apparent Emotion Rating
AI: artificial intelligence
APADEM: Apathy Scale for Institutionalized Patients with Dementia Nursing Home version
CERAD-K: Korean version of the Consortium to Establish a Registry for Alzheimer’s Disease
CHART: Craig Handicap Assessment and Reporting Technique
FIM: Functional Independence Measure
GDS: Global Deterioration Scale
GDSSF-K: Geriatric Depression Scale Short Form: Korean Version
K-CMAI: Korean version of the Cohen-Mansfield Agitation Inventory
MMSE: Mini Mental State Examination
MOCA-J: Japanese version of the Montreal Cognitive Assessment
NPI: Neuropsychiatric Inventory
OARS: Older Americans Resources and Services
PICOTS-SD: Population, Intervention, Comparison, Outcome, Time, Setting, and Study Design
PRISMA: Preferred Reporting Items for Systemic Reviews and Meta-Analyses
QUALID: Quality of Life in Late-stage Dementia
ROB: risk of bias
SAR: socially assistive robots
SIP: Sickness Impact Profile
SMCQ: Subjective Memory Complaint Questionnaire
TMIG-IC: Tokyo Metropolitan Institute of Gerontology-Index of Competence
