# Correction: The Effect of Cognitive Function Health Care Using Artificial Intelligence Robots for Older Adults: Systematic Review and Meta-analysis

**DOI:** 10.2196/42312

**Published:** 2022-09-14

**Authors:** Hocheol Lee, Min Ah Chung, Hyeji Kim, Eun Woo Nam

**Affiliations:** 1 Healthy City Research Center Yonsei University Wonju Republic of Korea; 2 Yonsei Global Health Center Yonsei University Wonju Republic of Korea; 3 Center of Evidence Based Medicine Institute of Convergence Science Yonsei University Wonju Republic of Korea; 4 Department of Health Administration College of Software and Digital Healthcare Convergence Yonsei University Wonju Republic of Korea

In “The Effect of Cognitive Function Health Care Using Artificial Intelligence Robots for Older Adults: Systematic Review and Meta-analysis” (JMIR Aging 2022;5(2):e38896), the authors made one addition.

The following information was added to the *Acknowledgments* section:

This research was supported by the Basic Science Research Program through the National Research Foundation of Korea (NRF) funded by the Ministry of Education (NRF-2021R1C1C2005464).

The correction will appear in the online version of the paper on the JMIR Publications website on September 14, 2022, together with the publication of this correction notice. Because this was made after submission to PubMed, PubMed Central, and other full-text repositories, the corrected article has also been resubmitted to those repositories.

