# Supplementary material for: The Effect of Cognitive Function Health Care Using Artificial Intelligence Robots for Older Adults: Systematic Review and Meta-analysis
Source: JMIR Aging. 2022 Jun 28;5(2):e38896. doi: 10.2196/38896 (PMC9277531; doi:10.2196/38896)
Supplement: Multimedia Appendix 1 [file aging_v5i2e38896_app1.docx]

Search keywords

(((((((Elderly) OR (Elderly People)) OR (older adults)) OR (older people)) OR (senior)) OR (Dementia)) OR (Alzheimer)) OR (Cognitive impairment) AND (((((((Robot) OR (AI robot)) OR (social assistive robot)) OR (social interactive robot)) OR (assistive robot)) OR (companion robot)) OR (robot interaction)) OR (health care robot) AND ((((((MMSE) OR (Mini-Mental State Examination)) OR (cognitive function)) OR (cognitive)) OR (cognitive impairment)) OR (cognitive disorder)) OR (mental health)
